# Comparison of the application of high-flow nasal oxygen with two different oxygen concentrations in infant and child laryngotracheal surgery

**DOI:** 10.3389/fmed.2023.1169345

**Published:** 2023-04-05

**Authors:** Jianxia Liu, Ling Xiong, Haisu Li, Min Du, Xue Ru, Ying Xu

**Affiliations:** ^1^Children's Hospital of Chongqing Medical University, Chongqing, China; ^2^National Clinical Research Center for Child Health and Disorders, Chongqing, China; ^3^Chongqing Key Laboratory of pediatrics, Chongqing, China; ^4^Ministry of Education Key Laboratory of Child Development and Critical Disorders, Chongqing, China

**Keywords:** high-flow nasal oxygenation, hyperoxia, oxygenation, STRIVE Hi, suspension laryngoscopic surgery

## Abstract

**Background:**

This study aimed to compare the use of the STRIVE Hi technique with 70 and 100% oxygen concentrations in children with 1st or 2nd degree laryngeal obstruction undergoing suspension laryngoscopic surgery.

**Methods:**

Children aged 1 month to 6 years scheduled for suspension laryngoscopic surgery with spontaneous respiration were randomly divided into the 70% oxygen concentration group (HFNO70% group) and the 100% oxygen concentration group (HFNO100% group). The data recorded for all the patients included age and sex, comorbidities, preoperative physiological status, methods of induction and maintenance of anesthesia, course of the disease and surgical options, and duration of operation. The primary endpoint was the lowest oxygen saturations during the surgery. The secondary endpoints included the partial pressure of oxygen PaO_2_, the arterial pressure of carbon dioxide PaCO_2_, the peak transcutaneous carbon dioxide PtcCO_2_, and the incidence of desaturation (defined as SpO_2_ < 90%) or hypercarbia (PtcCO_2_ > 65 mmHg).

**Results:**

A total of 80 children with 1st or 2nd degree laryngeal obstruction were included in the analysis. The median [IQR (range)] duration of spontaneous ventilation using STRIVE Hi was 52.5 [40–60 (30–170)]min and 62.5 [45–81 (20–200)]min in the HFNO 70% and HFNO 100% groups, respectively (*p* = 0.99); the lowest oxygen saturation recorded during the operation was 97.8 ± 2.1% and 96.8 ± 2.5%, respectively (*p* = 0.053); the mean PaO_2_ at the end of surgery was 184.6 ± 56.3 mmHg and 315.2 ± 101.3 mmHg, respectively (*p* < 0.001); and the peak transcutaneous CO_2_ was 58.0 ± 13.0 mmHg and 60.4 ± 10.9 mmHg, respectively (*p* = 0.373), despite a long operation time.

**Conclusion:**

STRIVE Hi had a positive effect on children undergoing tubeless laryngeal surgery with spontaneous ventilation, and for children with 1st or 2nd degree laryngeal obstruction, there was no significant difference in maintaining the intraoperative oxygenation between the 70 and 100% oxygen concentration groups. The 100% oxygen concentration group showed significant hyperoxia, which has been proven to be associated with multiple organ damage. Using a relatively lower oxygen concentration of 70% can effectively reduce the hazards associated with hyperoxia compared to 100% oxygen concentration.

**Clinical trial registration:**

[www.chictr.org.cn], identifier [CHICTR2200064500].

## Introduction

Laryngotracheal surgery in infants and children can be a very challenging surgical procedure. To obtain the optimal surgical conditions, endotracheal intubation is usually avoided to ensure that the view of the surgical field is not blocked by the endotracheal tube (1). Additionally, evaluation of the dynamics of the upper airways is usually required for an accurate diagnosis before the procedure, and further adjustments can be performed as necessary (2). Therefore, children are usually required to undergo surgery without tracheal intubation while preserving spontaneous breathing. An inadequate depth of anesthesia can cause laryngospasm, bronchospasm or barotrauma which may require muscle relaxation and possibly intubation (3). An excess depth of anesthesia can cause hypoventilation or apnea which may results in hypoxemia, necessitating rescue intubation or bag mask ventilation, and consequent interruption of the procedure and prolongation of the surgical time (4).

In recent years, the advantages of high flow nasal oxygen (HFNO) in maintaining oxygenation during anesthetic management in pediatric laryngotracheal surgery have been recognized. The STRIVE Hi technique, a tubeless, spontaneous respiration anesthesia technique that is used in inducing total intravenous anesthesia and administering high flow nasal oxygen (HFNO) (5), has the benefits of prolonging the safe apnea time and reducing the number of intermittent intubations (6).

There are several studies about the STRIVE Hi technique during laryngotracheal surgery (7, 8), and the oxygen concentration was commonly set as 100%. Excessively high oxygen concentrations can cause excessive hyperoxia in children, resulting in a series of complications, and the risk can specifically increase when the FiO2 exceeds 0.7 for several hours (9). Based on our early experience using STRIVE Hi in pediatric bronchoscopy, a 70% oxygen concentration was sufficient to complete the procedure, which was consistent with our pilot study of 22 patients with 1st and 2nd degree laryngeal obstruction undergoing suspension laryngoscopy surgery. So, we assumed that although the 100% oxygen concentration met the requirements for surgery, the relatively lower oxygen concentration of 70% would also meet the requirements of laryngotracheal surgery in children with a relatively low degree of laryngeal obstruction, thus reducing the risk of hyperoxia. To evaluate our hypothesis, we evaluated the use of STRIVE Hi with 70 and 100% oxygen concentrations in pediatric laryngotracheal surgery.

## Methods

This prospective, randomized, observational study was conducted in children with laryngotracheal diseases diagnosed and treated at the Children’s Hospital of Chongqing Medical University from October 8, 2022, to February 8, 2023. This study was approved by the Ethics Committee of the Children’s Hospital of Chongqing Medical University, which is a teaching hospital in Chongqing, China, with a tertiary surgical center for children.

The inclusion criteria were as follows: 1. Children who were scheduled for suspension laryngoscopy surgery, in which the surgeon requested tubeless anesthesia under spontaneous respiration in combination with STRIVE Hi, 2. Children aged 1 month to 6 years old; 3. Children with an American Society of Anesthesiologists (ASA) status of I-III, and 4. Children with combined 1st and 2nd degree laryngeal obstruction. The exclusion criteria were as follows: 1. Children with a skull fracture or facial fracture, 2. Children with an obvious obstruction of the nasal airflow due to changes in the nasal septum or nasal cavity, 3. Children who need neuromuscular block, tracheal intubation, or mechanical ventilation, 4. Children planned to undergo CO_2_ laser treatment, 5. Children with tracheotomy, and 6. Children with 3rd and 4th degree laryngeal obstruction.

The degrees of laryngeal obstruction are as follows: 1st degree: Mild dyspnoea during activities or crying; 2nd degree: Mild dyspnoea during crying and quiet time; 3rd degree: Dyspnoea with inspiratory triple concave sign at rest; 4th degree: Dyspnoea with inspiratory triple concave sign and cyanosis during quiet time. In our hospital, the degree of laryngeal obstruction is determined by a specialist and then recorded in the patient’s medical record as a diagnosis.

The children were randomly divided into the 70% oxygen concentration group (HFNO70% group) and the 100% oxygen concentration group (HFNO100% group) in a 1:1 ratio by using a computer-generated randomization program. The oxygen concentration was set by an anesthesiologist, and the screen was covered after setting. Group allocation was concealed, the patients and researchers who collected and analyzed the data were blinded to the allocation.

Both groups of patients were preoxygenated with 100% (5 L/min) oxygen *via* face mask using an anesthesia machine. After preoxygenation all patients were induced intravenously with midazolam 0.1 mg/kg, propofol 1.5–3 mg/kg and sufentanil 0.1–0.2 μg/kg to adequately balance the depth of anesthesia and maintain spontaneous ventilation. A high-flow nasal cannula appropriate for the patient’s age were secured, and oxygen was supplied by HFNO (Mindray SV300) at a rate of 2 l/kg/min. The only difference was that the oxygen concentration set in the two groups was 70% in the HFNO 70% group and 100% in the HFNO 100% group. After the intravenous anesthesia took effect, topical anesthesia was performed by spraying 1% lidocaine on the surface of the epiglottis, glottis and trachea through a nebulizer. Maintenance of anesthesia was modulated by an infusion of propofol at a rate of 5 mg/kg/h; if the children moved during the operation, propofol was injected alone at 1 mg/kg. Rescue oxygenation was defined as a rapid and continuous decline in SpO_2_ below 90% during the process, which required immediate interruption of the procedure to allow bag/mask ventilation or tracheal intubation as a rescue method.

The patients were monitored with a three-lead electrocardiogram, and noninvasive blood pressure, respiratory rate, pulse oximetry (SpO_2_) and transcutaneous CO_2_ (PtcCO_2_) were recorded. PtcCO_2_ was monitored with a sensor attached to the skin of the abdomen or anterior chest, and the sensor was connected to a monitor (SenTec digital transcutaneous monitor; SenTec AG) after calibration. Arterial blood gasses were checked at the beginning and end of surgery.

Data on the baseline characteristics of the included patients, preoperative comorbidities, symptoms, diagnosis, procedure, degree of laryngeal obstruction, durations of anesthesia and duration of PACU were collected. Parameters of oxygenation and ventilation, including SpO_2_, PtcCO_2_, PaCO_2_, and PaO_2_, were also recorded.

The primary endpoint was the lowest oxygen saturations during the surgery. The secondary endpoints included the partial pressure of oxygen PaO_2_, the arterial pressure of carbon dioxide PaCO_2_, the peak transcutaneous carbon dioxide PtcCO_2_, and the incidence of desaturation (defined as SpO_2_ < 90%) or hypercarbia (PtcCO_2_ > 65 mmHg).For calculate sample size, we conducted a pilot study of 22 patients with 1st and 2nd degree laryngeal obstruction undergoing suspension laryngoscopy surgery. In the pilot study, the lowest SpO_2_ was 95% during the surgery using HFNO with 100% oxygen concentration. To test the non-inferiority of HFNO with 70% oxygen concentration compared with HFNO with 100% oxygen concentration regarding the lowest SpO_2_ during surgery, 40 subjects per group were required for a non-inferiority margin of 3%.

SPSS v21.0 was used for the statistical analysis. The measurement data are expressed as the mean ± SD or median (M) and interquartile range (IQR). The mean differences between the groups were compared using the independent *t*-test or the Mann–Whitney U-test according to the normality of the variables. Differences in rates between groups were compared using a Pearson’s Chi-squared test and a Fishe’s exact test. Repeated-measures variables were compared using repeated-measures ANOVA. In all analyses, a *p* value <0.05 was taken to indicate statistical significance.

## Results

**Figure 1:**
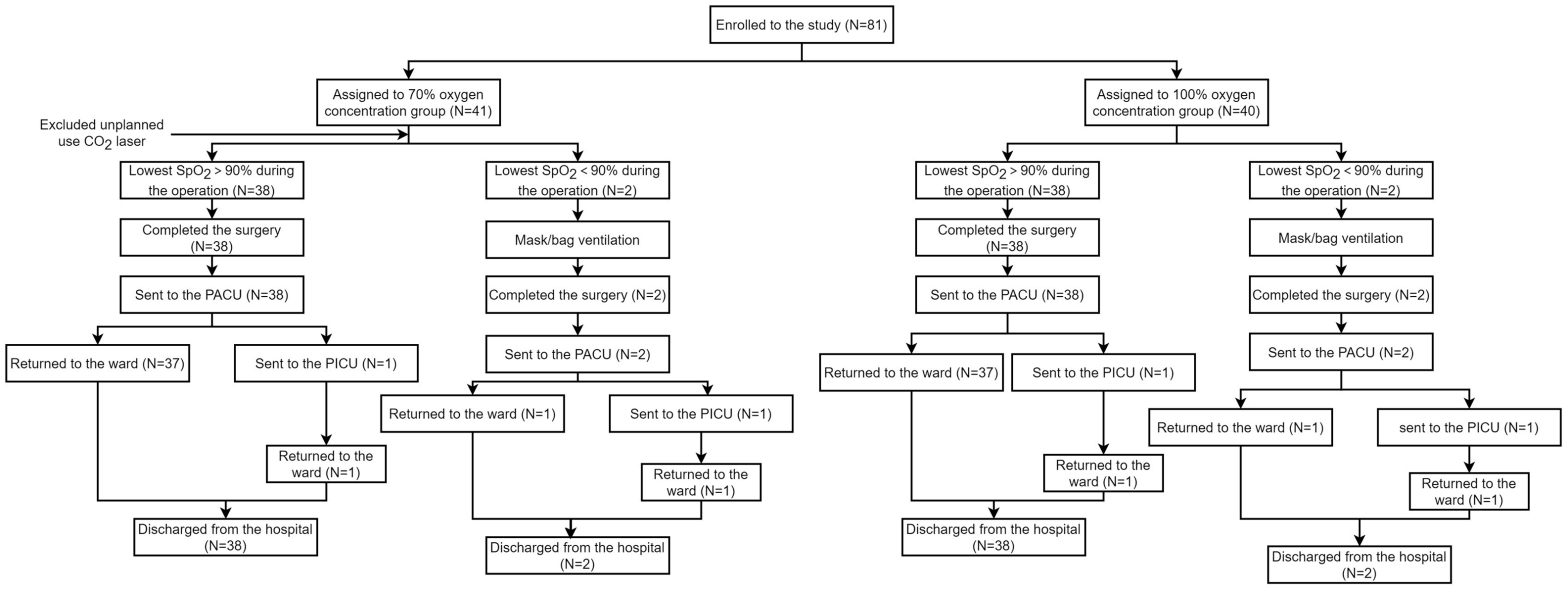
Consolidated Standards of Reporting Trials (CONSORT) diagram.

In our study, a total of 81 children with combined 1st and 2nd degree laryngeal obstruction underwent suspension laryngoscopy with intravenous and tracheal surface anesthesia, and the anesthesia was performed throughout the surgical process in a tubeless state. One patient was excluded due to the unplanned use of a CO_2_ laser (Figure 1). In total, 40 patients in the HFNO 70% group and 40 patients in the HFNO 100% group were included in the analysis.

The characteristics of the patients are presented in Table 1. The median age was 27.0 [11.5–38.3 (2–72)] months in the HFNO 70% group and 26.2 [11.8–36.0 (1–72)] months in the HFNO 100% group (*p* = 0.869). Among all the patients, 10 and 10 patients in the HFNO 70% and HFNO 100% groups were younger than 1 year old, and 2 and 3 patients were younger than 3 months, respectively. In the HFNO 70% group, 9 patients were diagnosed with 1st degree laryngeal obstruction, and 31 patients were diagnosed with 2nd degree laryngeal obstruction, compared to 10 and 30 patients in the corresponding 100% group. All patients presented with different airway and/or respiratory symptoms: 12 patients had laryngeal stridor, 23 patients were dyspneic, and 5 patients had obstructive sleep apnea syndrome in the HFNO 70% group, corresponding to 13, 20 and 7 patients in the HFNO 100% group. There were 6 patients who were treated with low-flow nasal cannula oxygen preoperatively in the HFNO 70% group and 7 in the HFNO 100% group in the ward. Some patients had preoperative complications, including stunting, congenital heart disease, pneumonia, and thoracic deformity. The clinical symptoms, diagnosis, procedure, and preoperative complications of the patients are shown in Table 2.

**Table 1:** Baseline characteristics of the subjects undergoing laryngeal procedure.

Variable	HFNO 70% (*n* = 40)	HFNO 100% (*n* = 40)	*p*-Value
Sex (female: male), *n* (%)	12 (30.0):28 (70.0)	10 (25.0):30 (75.0)	0.622
Weight (kg)	11.6 ± 6.5	10.4 ± 4.8	0.366
Age (month)	27.0 [11.5–38.3 (2–72)]	26.2 [11.8–36.0 (1–72)]	0.869
Age of onset			1
< 3 month	2 (5.0)	3 (7.5)	
< 1 year (except for <3 month)	8 (20.0)	7 (17.5)	
≥1 year and < 3 years	19 (47.5)	19 (47.5)	
≥3 years	11 (27.5)	11 (27.5)	
Laryngeal obstruction			0.793
I, *n* (%)	9 (22.5)	10 (25.0)	
II, *n* (%)	31(77.5)	30 (75.0)	
ASA			0.412
II, *n* (%)	7 (17.5)	10 (25.0)	
III, *n* (%)	33 (82.5)	30 (75.0)	

Data are the median (interquartile range), *n* (%), or mean (standard deviation). HFNO 70%, high-flow nasal cannula with 70% oxygen concentration. HFNO 100%, high-flow nasal cannula with 100% oxygen concentration.

**Table 2:** Clinical symptoms, diagnosis, procedure, and preoperative complications of the patients.

	HFNO 70% (*n* = 40)	HFNO 100% (*n* = 40)	*p*-Value
Respiratory symptom			0.896
Laryngeal stridor	12 (30)	13 (32.5)	
Dyspnoeic	23 (57.5)	20 (50.0)	
Obstructive sleep apnoea syndrome	5 (12.5)	7 (17.5)	
Used low-flow nasal cannula oxygen preoperation	6 (15.0)	7 (17.5)	
Diagnosis			0.959
Tracheomalacia	5 (12.5)	2 (5)	
Subglottic stenosis	10 (25)	10(25)	
Laryngeal papilloma	1 (2.5)	2 (5)	
Laryngeal cleft	4 (10)	5 (12.5)	
Epiglottic cyst	1 (2.5)	2 (5)	
laryngeal tumor	1 (2.5)	0(0)	
Bilateral vocal cord paralysis	10 (25)	9 (22.5)	
Laryngomalacia	7(17.5)	8 (20)	
Tracheoesophageal fistula	0(0)	1 (2.5)	
Laryngostenosis	1 (2.5)	1 (2.5)	
Procedure			0.990
Examination	40 (100)	40 (100)	
Ballooning		1 (2.5)	
Excision of epiglottis cyst	1 (2.5)	2 (5)	
Excision of neoplasm	9 (22.5)	10 (25)	
Excision of laryngeal tumors	1 (2.5)	2(5)	
Vocal cord abducent and fixed	6 (15)	8 (20)	
Excision of laryngeal papilloma	1 (2.5)	2 (5)	
Supraglottoplasty	5 (12.5)	6 (15)	
“T” tube placement	4 (10)	3 (7.5)	
Laryngeal Cleft repair	5 (12.5)	3 (7.5)	
Preoperative complications, *n* (%)			0.965
Stunting	7 (17.5)	5 (12.5)	
Congenital heart disease	5 (12.5)	3 (7.5)	
Pneumonia	11 (27.5)	10 (25)	
Thoracic deformity	2 (5)	1 (2.5)	

All the perioperative variables of oxygenation and ventilation in the patients who received oxygen supply using a high-flow nasal cannula with 70% or 100% oxygen concentration during the laryngeal procedure are shown in Table 3. For the primary outcome, SpO_2_ was compared between the two groups. The average preoperative SpO_2_ was 97 and 96% in the HFNO 70% group and HFNO 100% group, respectively (*p* = 0.053), corresponding to the lowest oxygen saturations during the operation was 95 and 95% in each group, respectively (*p* = 0.859). We also determined the number of patients whose lowest SpO_2_ was lower than 95%, and there was also no significant difference between the two groups. Only two patients in each group had the lowest oxygen saturation below 90%, and rescue interventions were implemented. The rescue interventions include jaw support and mask/bag ventilation. All 80 patients successfully completed the operation without tracheal intubation.

**Table 3:** Perioperative variables of oxygenation and ventilation.

Variable	HFNO 70% (*n* = 40)	HFNO 100% (*n* = 40)	*p*-Value
SpO_2_ (%)			
Preoperation	97.8 ± 2.1	96.8 ± 2.5	0.053
The lowest oxygen saturation during the operation. (%)	95.9 ± 6.0	95.7 ± 5.2	0.859
lowest SpO_2_ < 90%, *n* (%)	2	2	1
lowest SpO_2_ < 95%, (except for <90%), *n* (%)	5	6	1
lowest SpO_2_ > 95%, (except for <95%), *n* (%)	33	32	1
PaCO_2_ (mmHg)			
At the beginning of surgery	52.6 ± 9.3	51.9 ± 10.1	0.748
At the end of surgery	55.4 ± 11.6	55.4 ± 11.1	0.976
PaO_2_ (mmHg)			
At the beginning of surgery	199.4 ± 67.6	283.8 ± 111.2	0.000
At the end of surgery	184.6 ± 56.3	315.2 ± 101.3	0.000
Grade of hyperoxemia at the end of surgery			
GradeI 120 < PaO_2_ < 200 (mmHg), *n* (%)	30	3	0.000
GradeII 200 < PaO_2_ < 300 (mmHg), *n* (%)	8	14	0.000
GradeIII 300 < PaO_2_ < 400 (mmHg), *n* (%)	2	13	0.000
GradeIV 400 < PaO_2_ < 500 (mmHg), *n* (%)	0	8	0.000
GradeV >500 (mmHg), *n* (%)	0	2	0.000
Peak transcutaneous CO_2_ (mmHg)	58.0 ± 13.0	60.4 ± 10.9	0.373
Incidence of transcutaneous CO_2_ > 65 mmHg, *n* (%)	10 (25.0)	15 (37.5)	0.228
Rescue intervention, *n* (%)	2 (5)	2 (5)	1
The median [IQR (range)] duration of spontaneous ventilation using STRIVE Hi (min)	52.5 [40–60 (30–170)]	62.5 [45–81 (20–200)]	0.99
PACU stay (min)	47.5 ± 20.8	43.6 ± 18.4	0.05
ICU, *n* (%)	2(5)	2(5)	1

Data are the median (interquartile range), *n* (%), or mean (standard deviation).

For secondary outcome variables, the partial pressure of oxygen PaO_2_ was compared between the two groups at the beginning and the end of surgery. The HFNO100% group exhibited extremely severe hyperoxemia, and there was a statistically significant difference between the two groups (*p* < 0.001). Compared to 2 patients in HFNO70% group, 23 patients with a PaO_2_ over 300 mmHg in the HFNO 100% group developed severe hypoxemia (*p* < 0.001). We performed lung ultrasound in 9 patients with PaO_2_ above 300 mmHg and an operative time of more than 60 min. All of them presented with obvious atelectasis. Two of them were younger than 3 months and were sent to the ICU after surgery, and the rest were returned to the ward after awakening from anesthesia.

**Figure 2:**
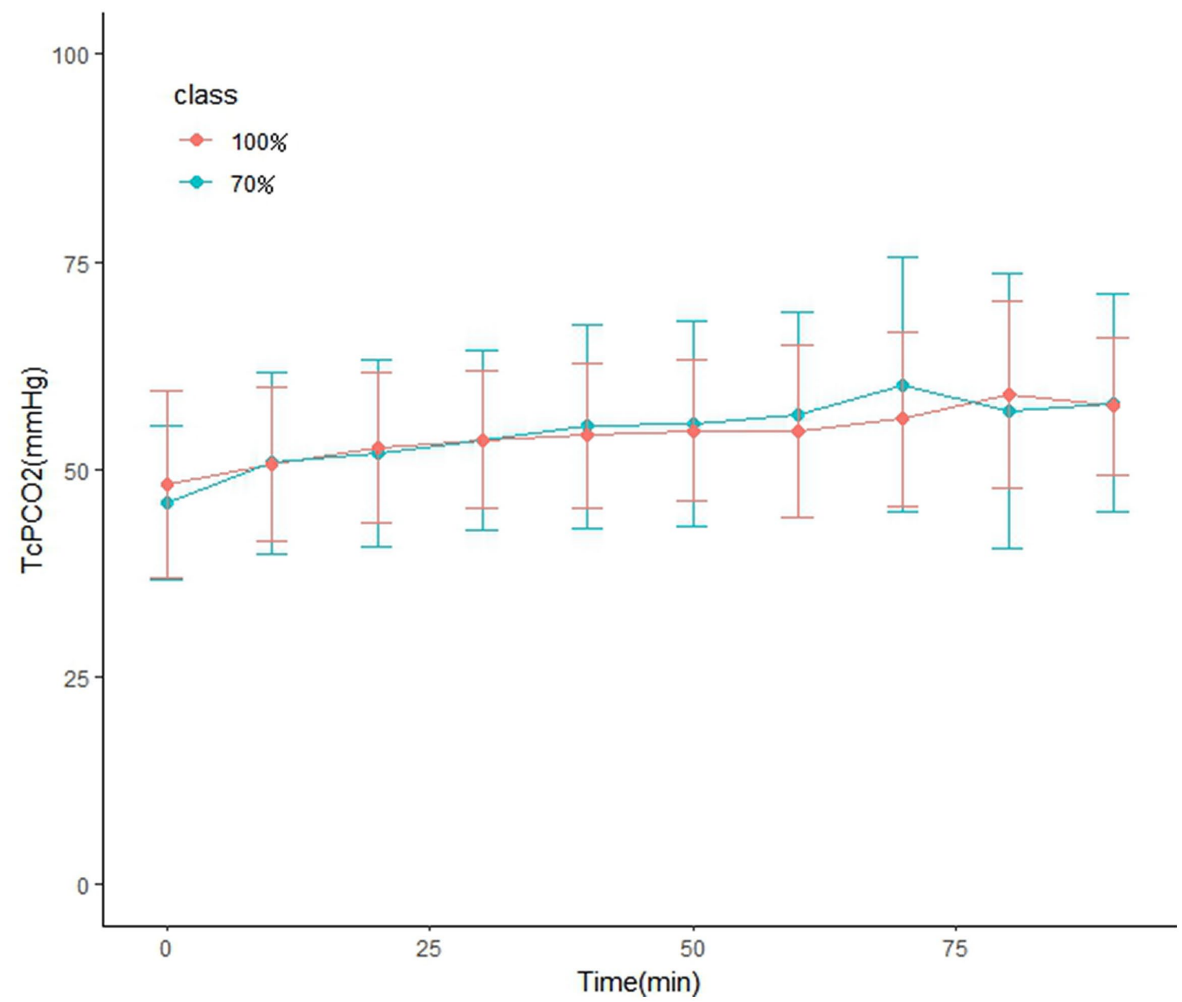
Transcutaneous carbon dioxide at baseline and at 10, 20, 30, 40, 50, 60, 70, 80, and 90 min during laryngeal procedure. Between-group comparisons were performed at each measurement time point using the independent *t*-test or Manne-Whitney *U*-test for multiple comparison. There was no difference between the two groups at any time point (*p* > 0.05).

The arterial pressure of carbon dioxide PaCO_2_ and the peak transcutaneous carbon dioxide PtcCO_2_ were compared between the two groups at the beginning and the end of surgery, and there was no significant difference between the two groups. For PtcCO_2_ at the beginning of the study and at 10, 20, 30, 40, 50, 60, 70, 80 and 90 min during the laryngeal procedure, there were no differences between the two groups at any time point (*p* > 0.05) (Figure 2). In our patients, the median duration (IQR) of spontaneous ventilation with STRIVE Hi was 52.5 [40–60 (30–170)] min in the HFNO 70% group and 62.5 [45–81 (20–200)] min in the HFNO 100% group (*p* = 0.99), which were rather long procedure times for laryngeal surgery under suspension laryngoscopy with spontaneous ventilation.

The average durations of PACU stay were 47.5 ± 20.8 min and 43.6 ± 18.4 min in the HFNO 70% and HFNO 100% groups, respectively (*p* = 0.05) (Table 3). Two patients in the HFNO70% group were transferred to the ICU for continued treatment after surgery; one of them was younger than 3 months old and had preoperative pneumonia and the other developed significant airway oedema after surgery. Two patients in the HFNO100% group were transferred to the ICU; one of them was younger than 3 months old and had postoperative atelectasis and the other was diagnosed as having a giant subglottic hemangioma. No pneumothorax, pneumomediastinum, or abdominal bloating was observed in any patient during the study period. All the patients underwent the operation without tracheal intubation and were finally discharged from the hospital.

## Discussion

The use of transnasal humidified rapid-inspiration ventilatory exchange (THRIVE) can significantly prolong the safe time of apnea oxygenation in apneic infants (10, 11). In this study, we used the STRIVE Hi technique, which is in principle the same oxygenation technique but is normally reserved for spontaneous breathing. The STRIVE Hi technique is a tubeless self-respiratory anesthesia method maintained intravenously, while heated and humidified oxygen is supplied by a high-flow nasal cannula (6, 12). The high-flow nasal cannula allows oxygen delivery without the presence of any equipment in the surgical field and airway. Additionally, the ability of this technique to maintain oxygenation even in the context of hypoventilation or apnea (4) allowed a deeper and satisfactory level of anesthesia in more stimulating procedures while reducing the risks of coughing, laryngospasm, and possibly rescue ventilation.

In our study, STRIVE Hi was used for children with 1st or 2nd degree laryngeal obstruction who underwent evaluation of tubeless airway dynamics and surgery with spontaneous ventilation. The median durations of the airway procedures were 52.5 and 62.5 min, and the maximum durations were 170 and 200 min in the HFNO 70% and HFNO 100% groups, respectively. There was no significant difference in maintaining the intraoperative oxygenation between the two groups. The average lowest oxygen saturation was 95 and 95% in each group. Only 2 patients in each group required a rescue intervention that included jaw support and mask/bag ventilation, and all patients successfully completed the operation without tracheal intubation. Although most of the current studies evaluating STRIVE Hi for laryngeal surgery used 100% oxygen concentration, we believe that after considering the hazards of hyperoxia (13, 14), it is worth considering appropriately and properly reducing the oxygen concentration in children with a relatively low degree of laryngeal obstruction.

The 100% oxygen concentration group (HFNO100% group) showed significant hyperoxia compared to the 70% oxygen concentration group (HFNO70% group). Exposure to a high or pure concentration of O_2_ for long periods causes pulmonary inflammation, acute hypoxic lung injury, atelectasis or O_2_ toxicity, with a higher and increasing risk specifically when FiO2 exceeds 0.7 for several hours (9). Hyperoxia is associated with increased morbidity in pediatric patients, (15) and the harmful effects depend on the degree of hyperoxia (16). Li and others (17) reported that acute hyperoxia rapidly provokes gut injury in a time- and dose-dependent manner and induces gut dysbiosis, and an innate immune response is involved in oxygen-induced gut injury. In our study, 23 patients with a PaO2 > 300 mmHg in the HFNO 100% group appeared to have severe hyperoxemia compared to 2 patients in the HFNO 70% group (*p* < 0.001); a higher degree of hyperoxemia can lead to more severe acute injury. We observed obvious atelectasis in 9 patients with a PaO_2_ greater than 300 mmHg and an operative time longer than 60 min, and 2 of them were sent to the ICU after surgery. We found in our study that children undergoing laryngeal surgery often had stunting, congenital heart disease, pulmonary inflammation and even respiratory failure preoperatively. For such patients, atelectasis may aggravate lung injury, prolong hospital stay, and even increase mortality (9). Therefore, for children with 1st or 2nd degree laryngeal obstruction, we propose using a relatively lower oxygen concentration of 70% to effectively reduce the hazards associated with hyperoxia compared to a 100% oxygen concentration.

A disadvantage of this technique is that it can mask apnea as the oxygen saturations are maintained for longer, and the concomitant increase in arterial CO_2_ can be greater than with traditional techniques (4). Although there is evidence that after bronchoscopy, acute postoperative hypercapnia with a CO_2_ level below 100 mmHg is not associated with adverse consequences and does not delay recovery in adults (18), we consider continuous CO2 monitoring essential because any elevations of CO_2_ need correction during surgery. In our study, the average PaCO_2_ was 52.6 ± 9.3 mmHg at the beginning and 55.4 ± 11.6 mmHg at the end of the surgery in the HFNO 70% group, while the average PaCO_2_ was 51.9 ± 10.1 mmHg at the beginning and 55.4 ± 11.1 mmHg at the end of the surgery in the HFNO 100% group. We also found that the PtcCO_2_ at baseline and at 10, 20, 30, 40, 50, 60, 70, 80 and 90 min during the laryngeal procedure was not different between the two groups at any time point. A.W.G. Booth reported that the rate of increase in ETCO_2_ was much lower in patients with STRIVE Hi with spontaneous breathing compared to patients with THRIVE (0.03 vs. 0.15 kPa·min-1), (5) which was consistent with our findings. We found that PtcCO_2_ did not rise as fast as reported in THRIVE despite a long operating time, which could be related to the spontaneous breathing technique; therefore, it may have a certain reduction effect on the rate of rise in CO_2_. Although there were 10 and 15 patients with peak PtcCO2 exceeding 65 mmHg in the HFNO 70% and HFNO 100% groups, respectively, there was no effect on the recovery time. Fifty-six children were sent back to the ward after waking up in the PACU, and 4 children were sent to the ICU because they were younger than 3 months or were complicated with preoperative pneumonia, postoperative atelectasis and severe airway oedema, respectively.

Compared to previous similar studies of tubeless laryngeal surgery with spontaneous ventilation in pediatric patients, the median durations of the airway procedures in our study were 52and 62 min and the maximum durations were 170 and 200 min in the HFNO 70% and HFNO 100% groups, respectively, which were much longer than that reported in previous articles. Our surgery types basically included the common pediatric laryngotracheal procedure under suspension laryngoscopy. The children included in our study were basically complicated with 1st or 2nd degree laryngeal obstruction before surgery, some were complicated with cardiopulmonary complications, and their age was the youngest compared to previous similar reports. Twenty patients in the two groups were younger than 1 year old, 5 patients were younger than 3 months, and the youngest patient was only 1 month. The STRIVE Hi technique had a positive effect on children of different ages undergoing tubeless laryngeal surgery with spontaneous ventilation despite a considerable duration of surgery in our study.

In previous reports, complications related to high-flow nasal oxygen developed in a 26-week-old infant with subcutaneous emphysema of the scalp associated with pneumo-orbitis (19). Three cases of serious air leak syndrome involving pneumothorax and pneumomediastinum were also reported in pediatric patients (20). A meta-analysis of randomized controlled trials have pointed out pneumothorax occurred in 17 of 2,498 children who received high-flow respiratory support (21). For newborns using high-flow nasal oxygen, we need to be more alert to the occurrence of pneumothorax. Two children undergoing cardiac surgery with HFNO developed abdominal distension, and the times that HFNO were given were 12 h and 24 h, respectively (22). Although none of these complications developed in our patients, risk factors due to high flow should be taken into consideration.

This study only included children with 1st or 2nd degree of laryngeal obstruction because we considered that a 70% oxygen concentration in STRIVE Hi would meet the requirements of laryngotracheal surgery for children with a relatively low degree of laryngeal obstruction. An excess oxygen concentration may increase the risk of hyperoxia. Children with 3rd or 4th degree laryngeal obstruction usually need auxiliary ventilation, endotracheal intubation, and even tracheotomy, especially for those receiving intraoperative ventilation through an endotracheal or tracheotomy tube. Children with 3rd or 4th degree laryngeal obstruction have higher requirements for oxygenation during operation, therefore, they are more likely to suffer from hypoxemia during operation and less likely to develop hyperoxia.

Laryngeal surgery requires deep anesthesia while maintaining adequate oxygenation, so more research is needed on the optimal flow and concentration of HFNO for laryngeal surgery. In our study, we only compared the use of two different oxygen concentrations with the STRIVE Hi technique in laryngeal surgery, and the optimal oxygen concentration is still unknown. Due to the high oxygenation requirements of laryngeal surgery, the oxygen concentration cannot be excessively reduced for avoiding the hazards associated with hyperoxia. More accurate monitoring methods are also required to evaluate the oxygenation state of children in real time in order to adjust the oxygen concentration in time to avoid hypoxia and hyperoxia.

## Conclusion

STRIVE Hi had a positive effect on children undergoing tubeless laryngeal surgery with spontaneous ventilation, and for children with 1st or 2nd degree laryngeal obstruction, there were no significant differences in maintaining intraoperative oxygenation between the 70% oxygen concentration group and the 100% oxygen concentration group. However, the 100% oxygen concentration group showed significant hyperoxia, which is associated with increased morbidity in pediatric patients. Therefore, we consider that using a relatively lower oxygen concentration of 70% can effectively reduce the hazards associated with hyperoxia compared to using a 100% oxygen concentration.

## Data availability statement

The original contributions presented in the study are included in the article/supplementary material, further inquiries can be directed to the corresponding author.

## Ethics statement

The studies involving human participants were reviewed and approved by the Ethics Committee of the Children’s Hospital of Chongqing Medical University. Written informed consent to participate in this study was provided by the participants’ legal guardian/next of kin.

## Author contributions

JL and YX designed the study and analyzed the data. HL and LX evaluated the manuscript. MD and XR performed the statistical measurement and analyzed the data. JL analyzed the data and wrote the paper. All authors have read and approved the final manuscript as submitted and agree to be accountable for all aspects of the work.

## Conflict of interest

The authors declare that the research was conducted in the absence of any commercial or financial relationships that could be construed as a potential conflict of interest.

## Publisher’s note

All claims expressed in this article are solely those of the authors and do not necessarily represent those of their affiliated organizations, or those of the publisher, the editors and the reviewers. Any product that may be evaluated in this article, or claim that may be made by its manufacturer, is not guaranteed or endorsed by the publisher.
